# A Case of Classic Raymond Syndrome

**DOI:** 10.1155/2012/583123

**Published:** 2012-08-09

**Authors:** Nicholas George Zaorsky, Jin Jun Luo

**Affiliations:** ^1^Department of Neurology, Temple University School of Medicine, 3401 North Broad Street, C525, Philadelphia, PA 19140, USA; ^2^Department of Pharmacology, Temple University School of Medicine, Philadelphia, PA 19140, USA

## Abstract

Classic Raymond syndrome consists of ipsilateral abducens impairment, contralateral central facial paresis, and contralateral hemiparesis. However, subsequent clinical observations argued on the presentation of facial involvement. To validate this entity, we present a case of classic Raymond syndrome with contralateral facial paresis. A 50 year-old man experienced acute onset of horizontal diplopia, left mouth drooling and left-sided weakness. Neurological examination showed he had right abducens nerve palsy, left-sided paresis of the lower part of the face and limbs, and left hyperreflexia. A brain MRI showed a subacute infarct in the right mid-pons. The findings were consistent with those of classic Raymond syndrome. To date, only a few cases of Raymond syndrome, commonly without facial involvement, have been reported. Our case is a validation of classic Raymond syndrome with contralateral facial paresis. We propose the concept of two types of Raymond syndrome: (1) the classic type, which may be produced by a lesion in the mid-pons involving the ipsilateral abducens fascicle and undecussated corticofacial and corticospinal fibers; and (2) the common type, which may be produced by a lesion involving the ipsilateral abducens fascicle and undecussated corticospinal fibers but sparing the corticofacial fibers.

## 1. Introduction

Classic Raymond syndrome, named after a French neurologist Fulgence Raymond, consists of ipsilateral abducens impairment, contralateral central facial paresis, and contralateral hemiparesis [1]. However, subsequent clinical observations argued on the presentation of facial involvement [2]. To validate this entity, we present a case of classic Raymond syndrome with contralateral facial paresis.

## 2. Case Report

**Figure 1:**
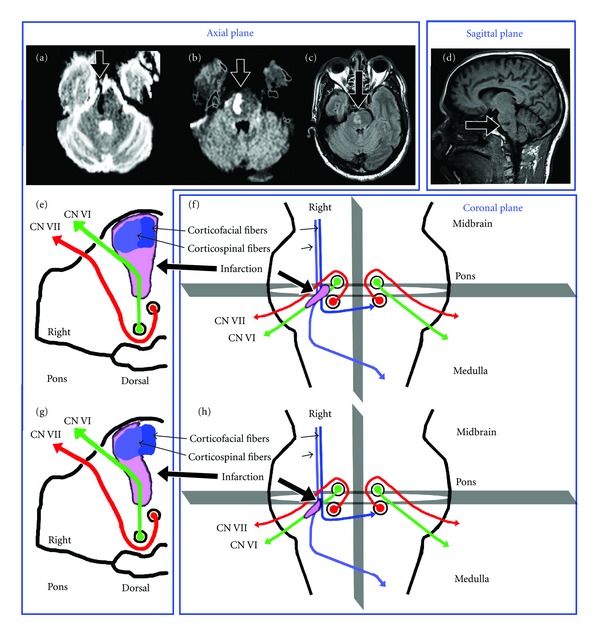
Brain MRI (3-Tesla) shows the axial apparent diffusion coefficient (a), diffusion weighted (b), fluid-attenuated inversion recovery (c), and sagittal T1 weighted (d) images. The schematic figures of the axial and coronal planes depict the classic ((e) and (f)) and common ((g) and (h)) types of Raymond syndrome.

 A 50-year-old man with hypertension, congestive heart failure, and polysubstance abuse (cocaine, cigarettes, alcohol, and marijuana) experienced three days of acute onset of horizontal diplopia, left mouth drooling, and left-sided weakness. On examination he had right abducens nerve palsy, left-sided central paresis of the lower part of the face and limbs, and left hyperreflexia. Pupils were equal, round, and reactive to light and accommodation. He did not exhibit ptosis. There was no muscle tenderness. Sensation was normal and intact. Cerebellar coordination exam was normal on the right but limited on the left due to weakness. The findings were consistent with those of classic Raymond syndrome [1] with facial nerve involvement. A brain MRI at 5 days after the onset of the symptoms showed a subacute infarct in the right midpons (Figures 1(a)–1(d)). The patient's symptoms were significantly improved with anticoagulative therapy in 3 days.

## 3. Discussion

Dr. Raymond first described a syphilitic woman with left abducens impairment, right central facial paresis, and right hemiparesis in 1896 [1]. Raymond hypothesized that a lesion in the lower medial pons damaged the abducens nerve and the nondecussated corticofacial and pyramidal fibers, but spared the more lateral facial nerve. However, after reviewing Raymond's original description, Wolfe disagreed with the hypothesis, citing the patient's development of a cerebral right hemiplegia, aphasia, and difficulty recognizing her husband's face [2], arguing that Raymond's explanation for the findings was unlikely; thus, not even Raymond's patient had “Raymond syndrome.” Subsequent clinical observations demonstrated that Raymond syndrome commonly seen in clinic was without contralateral facial involvement [3–5].

However, Sheth and colleagues recently reported a 55-year-old woman suffering from a sudden onset of right-sided weakness and diplopia with right central facial paresis and left abducens palsy due to a lacunar infarct in the base of the left medial caudal pons on neuroimaging studies [6]. Their observations were consistent with those of classic Raymond syndrome. Supportively, we presented an additional case of classic Raymond syndrome with contralateral facial paresis.

Raymond syndrome is an extremely rare neurologic entity. Its localization involves a restricted but sophisticated neural network resided in the medial lower pons among many other nuclei and neural fibers. Early neuroanatomic study demonstrated that the facial decussation occurs in the pons at the level of the facial nuclei [7], which provides evidence supporting the notion that a lesion in the basis of the medial caudal pons could produce Raymond syndrome [7, 8]. It has been suggested that the clinical manifestation of simultaneously ipsilateral abducens nerve palsy with contralateral central facial paresis seen in the classic Raymond syndrome results from a lesion in the pons involving the corticofacial decussation at the level of abducens nerve (Figures 1(e) and 1(f)) [6, 7], while a commonly seen Raymond syndrome may occur if corticofacial tract is spared (Figures 1(g) and 1(h)) [3–5]. Notably, a lesion in the more dorsal area may produce an isolated abducens nerve palsy [9] while the lesion in the ventral caudal pons may produce Millard-Gubler syndrome consisting of both ipsilateral abducens and facial paralysis, and contralateral hemiplegia [8, 10].

 Innovations in technology have dramatically expanded our knowledge of the functional and neuroanatomical structures. Urban and colleagues used transcranial magnetic stimulation to study the course of corticofacial projections in the human brainstem in patients with and without central facial paresis due to unifocal ischemic lesions of the brainstem [11]. In correlation with brain MRI, they identified corticofacial fibers may loop down into the ventral part of the upper medulla, cross the midline, and ascend in the dorsolateral medullary region to the facial nucleus in some patients. Their findings provide additional evidence suggesting that a contralateral central facial paresis may occur due to a unifocal lesion at the pontine base [11]. Interestingly, the corticofacial projection has also been identified in paramedial lemniscus as an aberrant pyramidal tract in the pons through the upper medulla [12].

To date, only a few cases of Raymond syndrome, commonly without facial involvement have been reported [3–5]. To our knowledge, the current case, with facial involvement, is the second validation of the classic Raymond syndrome after an extensive MEDLINE search [6]. We would, therefore, propose the concept of two types of Raymond syndrome: (1) the classic type, which may be produced by a lesion in the mid-pons involving the ipsilateral abducens fascicle and the non-decussated corticofacial and corticospinal fibers (Figures 1(e) and 1(f)); and (2) the common type, which may be produced by a lesion involving the ipsilateral abducens fascicle and non-decussated corticospinal while sparing the corticofacial fibers (Figures 1(g) and 1(h).
